# Nursing Students’ Satisfaction with Clinical Simulation: A Cross-Sectional Observational Study

**DOI:** 10.3390/nursrep14040231

**Published:** 2024-10-25

**Authors:** Juan Antonio Jiménez-Álvarez, María Dolores Guerra-Martín, Álvaro Borrallo-Riego

**Affiliations:** 1 Nursing Department, Osuna University School, 41640 Osuna, Spain; jajimenez@euosuna.org; 2 Nursing Department, Faculty of Nursing, Physiotherapy and Podiatry, University of Seville, 41009 Seville, Spain; aborrallo@us.es; 3 Institute of Biomedicine of Seville (IBiS), Antonio Maura Montaner Street, 41013 Seville, Spain

**Keywords:** education, nursing, personal satisfaction, simulation training, surveys and questionnaires, teaching

## Abstract

Clinical Simulation improves results in the students’ learning tests and allows for preserving acquired knowledge for longer periods of time, promoting more significant learning. This study was conducted to analyze Nursing students’ satisfaction with Clinical Simulation in three centres attached to a university from southern Spain. Methods: A quantitative, non-experimental and cross-sectional descriptive study was carried out. The students included were attending their third year of the Nursing undergraduate course and had already taken part in training sessions by means of Clinical Simulation. The Satisfaction Scale with High-Fidelity Clinical Simulation in Students (SSHF) was used for data collection. This scale has been validated and has 33 items grouped into eight factors. The SPSS software (version 28), was used for data analysis, establishing *p*-values < 0.05 for the statistically significant differences. Results: The participants were 180 students, with a mean age of 22.17 years old. Of them, 90.56% belonged to the female gender. A mean score of 3.82 out of 5 was obtained in the SSHF items. The items that obtained the highest scores were the following: benefits of Clinical Simulation as it relates theory with practise; possibility of learning based on the mistakes made; and comfort and respect while the sessions were developed. The item that obtained the lowest score was “timing for each simulation case”. We found significant differences in the results obtained according to each attached centre. Conclusions: The students showed high satisfaction levels regarding High-Fidelity Clinical Simulation in each of the three attached centres included in the study. Nevertheless, they stated the need to invest more time in Clinical Simulation sessions.

## 1. Introduction

The implementation of Clinical Simulation (CS) services is an indicator evidencing the quality of a teaching institution [1]. The students that take part in CS sessions attain more significant learning when compared to those that follow more traditional techniques. In addition, they improve their results in the learning tests and keep the acquired knowledge for longer periods of time [2,3,4,5,6].

CS is a teaching methodology that seeks to integrate students into an environment that simulates experiences from the clinical reality [7,8,9], and is ideal to encourage learning due to its safe and reproducible nature [10]. Given its methodological versatility and capability to monitor progress, CS constitutes an optimum means of mitigating the gap between theoretical learning and actual professional practise [11,12]. There are different types of CS: 1. Low-fidelity CS, which is generally used to acquire basic knowledge and skills in a concrete and specific human body area. 2. Medium-fidelity CS, which allows the representing of a body area along with fairly simple computer programs to develop a specific competence. 3. High-fidelity CS, where the devices offer the possibility of creating realistic clinical settings, allowing for the development of basic and advanced competences [13,14,15].

CS presents various advantages: on the one hand, it allows for creating immersive settings that enable developing technical dexterities and foster analysis ability for making and prioritizing critical decisions [16,17]. On the other hand, it promotes communicative competences, especially in multidisciplinary contexts, which helps improve the interaction with patients and collaborative work with the peers that take part in the simulated activity [12,18].

However, CS presents some disadvantages, such as the need to spend subsequent time to allow for the students’ reflection on the updates made (debriefing) [19], although some authors consider this reflection time very necessary to improve practise and achieve the highest quality [2]. Another inconvenience is the high costs that not all institutions can afford [20], although low- and medium-fidelity CS devices are by far less expensive than high-fidelity CS equipment [21].

CS has consolidated itself as a powerful methodology for training future nurses, allowing them to face actual clinical environments with greater self-confidence and competence [22,23]. In order to conduct real-time CS sessions, the students are asked to prioritize their actions and to make quick decisions, which promotes critical and clinical reasoning [16,24]. Consequently, better care quality is fostered, minimizing future errors and their possible negative repercussions [25].

The students’ satisfaction is one of the indicators to considerer when assessing the quality and efficiency of their learning [26,27,28]. High satisfaction levels among students increase their motivation, assisting in their acquisition of practical skills that are essential for their professional performance [29,30]. In other studies, CS enhances students’ satisfaction as long as the necessary equipment is available and the settings are designed based on their previous knowledge [31,32].

Assessing the students’ satisfaction with CS offers important information to analyze the extent to which their expectations are met, as well as to determine improvement areas. All of the aforementioned allows for designing more adequate settings and planning more effective CS sessions [16,26,33].

Various authors mention the need to foster research on the students’ satisfaction with CS to assess both the effectiveness of this teaching methodology and its effect when compared to others [34,35,36].

In the Spanish context, academic training for Nursing consists of a university course lasting four years with 240 European Credit Transfer System credits [37], where CS is used as a teaching methodology for the development of training and evaluative activities for a simulated demonstration of the competences acquired [16,38,39,40,41,42,43].

The objective of this study is to analyze Nursing students’ satisfaction with CS in three centres attached to a university from southern Spain.

## 2. Materials and Methods

### 2.1. Study Design and Participants

A quantitative, non-experimental and cross-sectional descriptive study was conducted [44]. To respond to the objective of this research, the following actions were carried out: 1. Characterization of the sample of students based on the independent variables, being gender, age and study centre; 2. Description of the students’ satisfaction level with CS according to the items and factors from the Satisfaction Scale with High-Fidelity Clinical Simulation in Students (SSHF); and 3. Comparison of the students’ satisfaction levels with CS across the different participating centres, according to the SSHF factors.

**Figure 1:**
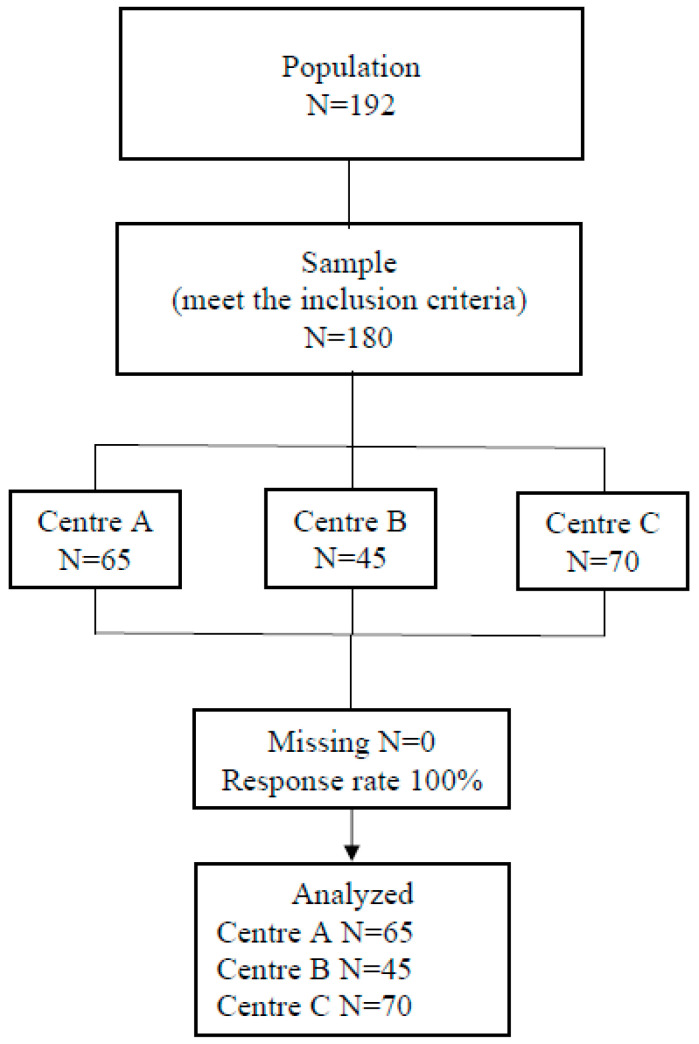
Participant flowchart. Source: the authors.

The students invited to take part in the study were those attending their third year of the Nursing course at three centres attached to a public university located in southern Spain, during the 2022–2023 academic year. The final sample comprised 180 students. Figure 1 shows the flowchart of participants.

The inclusion criteria were as follows: 1. Students enrolled in the third year of the Nursing course at any of the participating centres; and 2. Students that had already taken part in training sessions by means of CS. For the sample calculation, the total population of the three centres and the inclusion criteria were considered. Three clusters were subsequently formed, one for each center [45].

### 2.2. Instrument

To assess the Nursing students’ satisfaction with the CS service, the Satisfaction Scale with High-Fidelity Clinical Simulation in Students (SSHF) was used, which was validated in Spanish by a multidisciplinary team comprising experts, obtaining a Cronbach’s alpha of α = 0.92 [38]. SSHF has 33 items with a Likert-type scale that offer five answer options: 1. Strongly disagree. 2. Disagree. 3. Indifferent. 4. Agree. 5. Strongly agree. Options 1 and 2 correspond to low satisfaction, and options 4 and 5 represent high satisfaction, except for item 13, where scores of 4 and 5 indicate low satisfaction. The items from this scale are divided into eight factors to assess satisfaction: 1. Simulation utility. 2. Characteristics of cases and applications. 3. Communication. 4. Self-reflection on performance. 5. Self-confidence. 6. Relation between theory and practise. 7. Facilities and equipment. 8. Negative aspects of simulation (this last factor only includes item 13) [16,38,39]. Table 1 shows the SSHF scale, with its items and associated factors.

### 2.3. Data Collection

The scale was administered to students who had taken the “Life Support” subject, with a total of ten sessions of two hours each. The three centres were located in different geographical locations but had similar characteristics in terms of students, teaching plans and facilities/equipment. In terms of facilities, all three centres have rooms to simulate hospital or home care environments, as well as a room for debriefing. In terms of equipment, these centres have task trainers and mannequins, including full-body ones and interactive electronic devices.

For data collection, a questionnaire was applied between December 2022 and February 2023; it included questions to collect the sociodemographic variables (gender, age and origin attached centre) and the SSHF questions.

The questionnaire was applied to the students in paper format at each of the attached centres, after the school hours. The training was carried out by previously contacting the coordinators of the academic disciplines, who taught part of their lessons with the CS methodology. After obtaining the coordinators’ approval, the location and time for data collection was defined. The students were duly informed verbally and in writing (information sheet) about the study objectives and the voluntary nature of their participation. In addition, they were asked to provide their informed consent to take part in this study.

### 2.4. Data Analysis

The SPSS software, version 28 (IBM Corporation, Armonk, NY, USA) was used for the statistical analysis. A descriptive analysis of the answer frequencies was performed for each item, calculating the mean values and standard deviations for each scale item and for all eight associated factors. For the associated factors, the medians and interquartile ranges were also calculated. To calculate the scores for the items and factors, the values given on the Likert scale were considered. The Kruskal–Wallis test was used to compare the SSHF items according to each attached centre. Non-parametric statistics were used after applying the Kolmogorov–Smirnov test and obtaining that the data sample did not follow a normal distribution. *p*-values < 0.05 were established as statistically significant differences [45].

### 2.5. Ethical Considerations

This study was approved by the Andalusian Biomedical Research Ethics Committee (Date: 24 November 2022, Code: 1310-N-22) and was conducted according to the criteria set forth in the Declaration of Helsinki [46]. Prior to initiating the research, the SSHF validation study authors were asked due permission, obtaining their consent. In addition, authorization was obtained from the directors of the participating attached centres before data collection.

Participation was voluntary, and the students were informed that they may withdraw from the study at any time. They were assured that participating in this study would mean no risk to, nor exert any influence on, their grades.

## 3. Results

### 3.1. Characteristics of the Students

The sample was composed of 180 students attending their third year of the Nursing course in three centres [Centre A: 65 (36.11%); Centre B: 45 (25%); Centre C: 70 (38.89%)]. The mean age of the sample was 22.17 years old (SD = 3.29; range = 19–45), with 90.56% (n = 163) being women; both values were similar for all participants. Table 2 describes the mean age, standard deviation, age range and percentage of women per center.

### 3.2. Students’ Satisfaction Levels Regarding Clinical Simulation According to the SSHF Items and Factors

As a first step, the students’ Satisfaction Level with CS is described according to the SSHF items and factors in the entire sample, including all three participating centres. In relation to the items, a mean score of 3.82 out of 5 was obtained. Item 14 (CS is helpful as it relates theory with practise) was the one that reached the highest scores. High scores were also obtained in item 6 (I felt comfortable and respected during the sessions) and in item 31 (CS will allow me to learn from the mistakes I made). To the contrary, item 4 (Is timing for each simulation case adequate?) and item 22 (It improves communication with the family) obtained the lowest scores. Table 3 shows the results from the entire sample in each SSHF item.

Regarding the results of the SSHF factors in the entire sample, the mean of the items included in each factor was calculated. In this sense, the factors that obtained the highest mean scores were Factor 2 (Characteristics of cases and applications), Factor 8 (Negative aspects of simulation) and Factor 4 (Self-reflection on performance), with this latter one obtaining the highest score: 4.09. To the contrary, Factor 6 (Relation between theory and practise) and item 7 (Facilities and equipment) obtained the lowest scores: 3.65 and 3.72, respectively.

As a second step, the students’ Satisfaction Level with CS is described according to the SSHF items and factors in each participating centre. As for the items, in Centre A, item 6 (I felt comfortable and respected during the sessions) obtained the highest score. In Centre B, items 2 (Are the case simulation objectives clear?), 14 (Simulation is helpful as it relates theory with practise) and 31 (Simulation will allow me to learn from the mistakes I made) were equal in reaching the highest scores. Item 14 also obtained the highest score in Centre C. Item 4 (Is timing for each simulation case adequate?) was the one with the lowest score in all three centres. Table 3 shows the results according to the SSHF items in each centre.

Regarding the results of the SSHF factors, the following values were obtained in each centre: In Centres A and B, Factor 4 (Self-reflection on performance) was the one that obtained the highest mean score. In Centre C, Factor 8 (Negative aspects of simulation) obtained the highest score. On the other hand, the lowest scores were obtained in the following cases: Centre A, Factor 1 (Simulation utility); Centre B, Factor 7 (Facilities and equipment); Centre C, Factor 6 (Relation between theory and practise). Regarding the medians, a value of 4.0 was obtained in all factors, regardless of the center. There was only one exception, being factor 8 of center C, with a median of 4.5. Table 4 includes the mean values, standard deviations, medians and interquartile ranges corresponding to the SSHF factors in each centre.

### 3.3. Comparison of the Students’ Satisfaction Levels with Clinical Simulation Across the Different Participating Centres, According to the SSHF Factors

The following results were obtained when comparing the students’ satisfaction levels with CS across the different centres according to the SSHF factors: on the one hand, Centre C reached the highest average range across all factors. On the other hand, statistically significant differences were detected: 1. In factors 3, 5, 7 and 8, differences were noticed between Centre B and Centre C, with better scores obtained in Centre C in all cases. 2. In factors 1 and 8, between centres A and C, with better results again obtained in the latter. 3. In Factor 7 between centres A and B, with better results in the former. Likewise, in Factors 2, 4 and 6, no significant differences were detected among the centres (Table 5).

## 4. Discussion

The data obtained in our study show that the students have expressed high satisfaction levels regarding CS as a methodology. These results are in line with other studies about Nursing students’ satisfaction with CS [9,11].

The students have mostly stated agreement that CS helps relate theory with practise; this item is the best assessed in the entire sample and obtains the highest score in Centre C. Other authors agree with this result. Baptista et al. [47] and Sánchez Maldonado et al. [9] describe the benefit that CS contributes to the students when the theoretical knowledge learned in the classroom is put into practise. In addition, Moreno-Cámara et al. [48] assert that CS allows students to act and think as if it were a real-life case, which helps foster understanding of the patient according to the case, and applying the theory learned to the practise in order to seek the best care possible.

The students expressed high satisfaction levels regarding the comfort and respect they felt during the CS sessions, which is in line with other authors [39,42]. Likewise, other authors set forth the importance of respect among the students and their comfort during the CS sessions [49,50,51].

Most of the students proved to be highly satisfied with the capabilities provided by CS, as it allows them to make mistakes and learn from them, which is in consonance with other studies [33,40]. Some authors mention that, if complemented with positive feedback by the teacher, the possible errors made during CS allow for the promotion of more significant learning without endangering patients’ lives [30].

The students stated feeling scarcely satisfied with the time devoted to the CS sessions. The same happens in other studies, such as those conducted by Sánchez Maldonado et al. [9] and by Astudillo-Araya et al. [52]. In this sense, Horsley et al. [53] consider time as an important factor to be taken into account in CS. These authors recommend adapting the simulation time to the number of students and their knowledge level. Likewise, low satisfaction levels were also stated regarding the usefulness of CS in improving communication with the family. These findings coincide with various authors, who assert that these values can be influenced by the absence of specific objectives when addressing this aspect in the simulation designs, so they must be incorporated [40,41].

In some of the centres, the students expressed high satisfaction in relation to the clarity of the simulation objectives. In this sense, The International Nursing Association for Clinical Simulation and Learning Standards Committee [54] indicates that it is indispensable to deal with the CS session objectives clearly during debriefing for the students’ proper learning.

Referring to the factors, the students stated that they were satisfied with the characteristics of the cases presented and their applications. Some authors, like Ruiz Vera and Martini [55], highlight the importance of designing CS settings that are suitable for the learning objectives or strategies that the students have to learn. Olaussen et al. [32] coincidentally mentioned the need for these settings to be adapted to the students’ knowledge level. These factors exert an effect on the students’ stress and anxiety levels while the CS sessions are developed, with an impact on their satisfaction [35,56].

Another factor that achieved high satisfaction rates was the one related to Self-reflection on performance. In this sense, other authors such as Ming Chow et al. [35] describe the importance of debriefing after a CS session, which allows the students to reflect both on their performance during the simulation and on the possible improvements that they might apply in their practise. In addition, Boostel et al. [57] highlights the usefulness of CS as an opportunity to place the students in unprecedented settings while they are developing their clinical practises.

The students indicated low satisfaction levels for the factor that made a reference to facilities and equipment. Some authors, such as Li et al. [31], indicate that a lack of sufficient equipment can impose the need to turn some students into observers, leading to a reduction in their satisfaction levels when compared to those that are active participants in the setting [58]. In this sense, proper planning of the simulation scenario is necessary, providing a clear description of it (people who will participate and necessary equipment and facilities) [2]. In addition, other authors, such as Cura et al. [20], point out that the students who are offered high-fidelity CS achieve greater satisfaction levels than those who participate in low- or medium-fidelity CS sessions.

Some students reported low satisfaction regarding usefulness of the simulations. Nevertheless, other authors describe the utility of CS as a methodology that allows students to make mistakes and reflect on them, in addition to increasing the students’ responsibility for their own learning in a safe environment closer to reality [17,59].

In general, the students agree that CS increases their concern about the competences inherent to nurses, with higher results found in Centre C. These findings are in line with those of various authors [39,41]. CS promotes the acquisition of competencies and skills, such as empathy and leadership development. This implies the possibility of experiencing situations that do not commonly occur in practise, which could increase the student’s initial concern. However, CS allows the creation of safe environments for students to acquire these skills and abilities, with the possibility of making mistakes and without prejudice to patients [2,3].

### Limitations

Several limitations were found in the current study. Firstly, despite including several study centres, the sample size was limited both in number and in geographical dispersion. It is for this reason that future studies should consider the possibility of using larger samples that allow generalization of the data obtained. Secondly, data collection was conducted by means of a self-applied questionnaire. This procedure may have caused biases based on each student’s understanding of the information requested. Thirdly, no long-term assessment was carried out, which is one reason why the possibility of conducting longitudinal studies about this theme in the future should be considered. Fourthly, repeating students have not been considered as an exclusion criterion, which may influence the results. Finally, it should be noted that the results of this research provide important information that can be taken into account by managers of university Nursing centres, in order to implement improvement strategies in Clinical Simulation services.

## 5. Conclusions

In general, the Nursing students proved to be highly satisfied with CS in the entire sample. They showed themselves very satisfied with the ability of CS to integrate theoretical and practical knowledge, as well as to be able to learn from their own mistakes. Likewise, they were extremely satisfied with the characteristics of the cases and their applications, as well as with their self-reflection on their own performance. However, they indicated lower satisfaction levels regarding the facilities and equipment available, in addition to requesting that more time should be devoted to the CS sessions. Finally, it should be noted that, in the comparison among the teaching centres, Centre C is the one that obtained the best results in all the SSHF factors.

## Figures and Tables

**Table 1:** Scale with High-Fidelity Clinical Simulation.

Factors	Conceptual Definition	Items
1. Simulation utility	Suitability of CS as a tool that eases learning.	7: Simulation is useful to assess the clinical status of a patient. 10: Simulation will improve my ability to provide care to my patients. 12: Simulation will improve communication and the ability to work with the team. 15: Simulation allows us to plan patient’s care effectively. 16: Simulation will improve my technical skills. 17: Simulation will reinforce my critical thinking and decision-making. 18: Simulation will help me assess the patient’s condition. 19: This experience will help me prioritize care. 21: Simulation improves communication with the team. 22: It improves communication with the family. 23: It improves communication with the patient. 26: Simulation will improve my clinical competence. 31: Simulation will allow me to learn from the mistakes I made.
2. Characteristics of cases and applications	Suitability of the clinical settings developed in relation to the students’ own characteristics.	2: Are the simulation objectives clear? 5: The degree of difficulty of the cases is adequate to my knowledge. 6: I felt comfortable and respected during the sessions. 8: Simulation practice lets you learn how to avoid making mistakes.
3. Communication	Communication tasks that are carried out while developing CS sessions.	27: The teacher always gives constructive feedback after each simulation session. 28: Debriefing at the end of the session allows reflecting on the cases. 29: Debriefing at the end of the session helps to correct mistakes.
4. Self-reflection on performance	CS application in clinical practices.	9: Simulation will help me set priorities for action in clinical situations. 11: Simulation will make me think about my next clinical practice. 32: Simulation is useful in the practice.
5. self-confidence	Repercussion level of the CS sessions on the students’ self-confidence.	20: Simulation promotes self-confidence. 24: Simulation will improve my self-confidence. 33: I will meet my learning expectations with these sessions.
6. Relation between theory and practice	The ability of CS to act as a bridge between theoretical and practical knowledge.	3: Do the cases recreate real situations? 4: Is timing for each simulation case adequate? 14: Simulation is helpful as it relates theory with practice.
7. Facilities and equipment	CS and the students’ ability to create an immersive clinical setting.	1: Do you think that the simulation classrooms where the cases are developed are real? 25: Yom can “lose your cool” with some cases during the simulation sessions. 30: I already know the theoretical part of the cases.
8. Negative aspects of simulation	Characteristics of CS that might suppose negative repercussions.	13: Simulation will make me more worried about the competences that a graduated nurse should have.

Source: Alconero-Camarero et al. [16,38,39].

**Table 2:** Mean age, standard deviation, age range and percentage of women per center.

Centres	Mean Age (Standard Deviation)	Age Range	% of Women (N)
Centre A	22.75 (4.84)	19–40	92.31 (60)
Centre B	21.74 (2.57)	20–33	89.13 (41)
Centre C	21.94 (1.48)	20–25	88.57 (62)

Source: the authors.

**Table 3:** Results according to the Scale with High-Fidelity Clinical Simulation items in the entire sample and in the each of the centres.

Items	N (%)												
	Entire Sample				Centre A			Centre B			Centre C		
	S. 1 y 2	S. 3	S. 4 y 5	$\bar{X}$ (SD)	S. 1 y 2	S. 3	S. 4 y 5	S. 1 y 2	S. 3	S. 4 y 5	S. 1 y 2	S. 3	S. 4 y 5
1	41 (22.78)	19 (10.56)	120 (66.67)	3.57 (±0.98)	10 (15.38)	2 (3.308)	53 (81.54)	18 (40)	7 (15.56)	20 (44.44)	13 (18.57)	10 (14.29)	47 (67.15)
2	4 (2.22)	19 (10.56)	157 (87.22)	4.1 (±0.66)	3 (4.61)	9 (13.85)	53 (81.54)	1 (2.22)	3 (6.66)	41 (91.11)	0 (0)	7 (10)	63 (90)
3	20 (11.17)	25 (13.89)	134 (74.86)	3.94 (±0.99)	7 (10.77)	5 (7.69)	53 (81.54)	9 (20)	7 (15.56)	29 (64.45)	4 (5.71)	12 (17.14)	53 (75.71)
4	107 (60.11)	21 (11.67)	50 (28.09)	2.62 (±1.1)	38 (58.46)	5 (7.69)	21 (32.31)	21 (46.66)	12 (26.67)	11 (24.44)	44 (62.86)	4 (5.71)	22 (31.43)
5	10 (5.62)	33 (18.33)	135 (75.84)	3.89 (±0.79)	3 (4.61)	11 (16.92)	51 (58.46)	2 (4.44)	6 (13.33)	36 (80)	5 (7.14)	16 (22.86)	49 (70)
6	0 (0)	12 (6.67)	168 (93.33)	4.42 (±0.61)	0 (0)	2 (3.08)	63 (96.92)	0 (0)	5 (11.11)	40 (88.89)	0 (0)	5 (7.14)	65 (92.86)
7	23 (12.78)	22 (12.22)	135 (75)	3.92 (±1.04)	8 (12.31)	9 (13.85)	48 (73.85)	8 (17.77)	7 (15.56)	30 (66.67)	7 (10)	5 (7.14)	58 (82.86)
8	42 (23.33)	36 (20)	102 (56.67)	3.45 (±1.1)	19 (29.23)	10 (15.38)	36 (55.39)	8 (17.78)	10 (22.22)	27 (60)	14 (20)	16 (22.86)	40 (57.14)
9	11 (6.11)	23 (12.78)	146 (81.11)	4.04 (±0.87)	8 (12.3)	8 (12.31)	49 (75.38)	2 (4.44)	8 (17.78)	35 (77.78)	1 (1.43)	6 (8.57)	63 (90)
10	13 (7.22)	21 (11.67)	146 (81.11)	4.04 (±0.9)	7 (10.76)	9 (13.85)	49 (75.38)	2 (4.44)	8 (17.78)	35 (77.78)	4 (5.71)	4 (5.71)	62 (88.57)
11	11 (6.11)	20 (11.11)	149 (82.78)	4.04 (±0.84)	5 (7.69)	5 (7.69)	55 (84.61)	1 (2.22)	5 (11.11)	39 (86.67)	5 (7.14)	10 (14.29)	55 (78.57)
12	17 (9.44)	28 (15.56)	135 (75)	3.84 (±0.87)	10 (15.39)	7 (10.77)	48 (73.84)	6 (13.33)	11 (24.44)	28 (62.23)	1 (1.43)	9 (12.86)	60 (85.71)
13	16 (8.94)	27 (1)	136 (75.98)	3.99 (±0.96)	8 (12.31)	17 (26.15)	39 (60)	4 (8.88)	5 (11.11)	36 (80)	4 (5.71)	4 (5.71)	62 (88.57)
14	1 (0.56)	9 (5)	168 (94.38)	4.4 (±0.61)	0 (0)	4 (6.15)	61 (93.85)	0 (0)	4 (8.89)	41 (91.11)	1 (1.43)	1 (1.43)	66 (94.29)
15	17 (9.6)	40 (22.22)	120 (67.8)	3.74 (±0.86)	6 (9.23)	16 (24.62)	40 (61.53)	6 (13.33)	8 (17.78)	31 (68.89)	5 (7.14)	16 (22.86)	49 (70)
16	20 (11.17)	28 (15.56)	131 (73.18)	3.9 (±0.96)	9 (13.85)	7 (10.77)	48 (73.84)	5 (11.11)	10 (22.22)	30 (66.67)	6 (8.57)	11 (15.71)	53 (75.71)
17	11 (6.21)	28 (15.56)	138 (77.97)	3.93 (±0.81)	8 (12.31)	14 (21.54)	41 (63.08)	2 (4.44)	4 (8.89)	39 (86.67)	1 (1.43)	9 (12.86)	59 (84.28)
18	29 (16.2)	39 (21.6)	111 (62.01)	3.63 (±0.97)	13 (20)	16 (24.62)	35 (53.85)	7 (15.56)	10 (22.22)	28 (62.22)	8 (11.43)	13 (18.57)	49 (70)
19	14 (7.82)	34 (18.89)	131 (73.18)	3.87 (±0.85)	8 (12.31)	16 (24.62)	40 (61.54)	4 (8.89)	6 (13.33)	35 (77.78)	2 (2.86)	11 (15.71)	57 (81.69)
20	18 (10.06)	37 (20.56)	124 (69.27)	3.84 (±0.95)	7 (10.77)	13 (20)	44 (67.69)	9 (20)	10 (22.22)	26 (57.77)	2 (2.86)	14 (20)	54 (77.15)
21	21 (11.67)	40 (22.22)	119 (66.11)	3.78 (±0.99)	12 (18.46)	13 (20)	40 (61.54)	5 (11.11)	13 (28.89)	27 (60)	3 (4.29)	14 (20)	53 (75.71)
22	66 (36.67)	67 (37.22)	47 (26.11)	2.82 (±1.09)	21 (32.31)	28 (43.08)	16 (24.61)	16 (35.55)	18 (40)	11 (24.44)	28 (40)	21 (30)	21 (30)
23	52 (29.71)	50 (27.78)	73 (41.71)	3.13 (±1.07)	19 (29.23)	24 (36.92)	21 (32.31)	14 (31.1)	12 (26.67)	17 (37.78)	19 (27.14)	13 (18.57)	36 (51.43)
24	23 (13.07)	35 (19.44)	118 (67.05)	3.77 (±1)	11 (16.93)	11 (16.92)	43 (66.16)	8 (17.78)	10 (22.22)	25 (55.56)	4 (5.71)	14 (20)	50 (71.43)
25	26 (14.77)	26 (14.44)	124 (70.45)	3.77 (±1.02)	11 (16.93)	16 (24.62)	38 (58.46)	10 (22.22)	6 (13.33)	27 (60)	5 (7.14)	4 (5.71)	59 (84.28)
26	9 (5.14)	18 (10)	148 (84.57)	4.01 (±0.72)	4 (6.15)	8 (12.31)	53 (81.54)	4 (8.89)	3 (6.66)	35 (77.78)	1 (1.43)	7 (10)	60 (85.71)
27	18 (10.23)	26 (14.44)	132 (75)	3.83 (±0.9)	5 (7.69)	13 (20)	47 (72.31)	9 (20)	9 (20)	25 (55.56)	4 (5.71)	4 (5.71)	60 (85.71)
28	6 (3.41)	55 (30.56)	115 (65.34)	3.74 (±0.71)	2 (3.08)	18 (27.69)	45 (69.23)	3 (6.66)	18 (40)	22 (48.89)	1 (1.43)	19 (27.14)	48 (68.57)
29	8 (4.57)	40 (22.22)	127 (72.57)	3.86 (±0.76)	5 (7.69)	8 (12.31)	52 (80)	2 (4.44)	16 (35.56)	25 (55.56)	1 (1.43)	16 (22.86)	50 (71.43)
30	18 (10.23)	28 (15.56)	130 (73.86)	3.83 (±0.91)	3 (4.61)	12 (18.46)	50 (76.92)	5 (11.11)	5 (11.11)	33 (73.33)	10 (14.29)	11 (15.71)	47 (67.14)
31	4 (2.29)	8 (4.44)	163 (93.14)	4.21 (±0.66)	3 (4.62)	4 (6.15)	58 (89.23)	0 (0)	2 (4.44)	41 (91.11)	1 (1.43)	2 (2.86)	64 (91.43)
32	8 (4.57)	14 (7.78)	153 (87.43)	4.18 (±0.83)	4 (6.16)	9 (13.85)	52 (80)	3 (6.67)	2 (4.44)	37 (82.22)	1 (1.43)	3 (4.29)	64 (91.43)
33	18 (10.23)	28 (15.56)	130 (73.86)	3.88 (±0.93)	7 (10.77)	11 (16.92)	47 (72.31)	10 (22.22)	7 (15.56)	26 (57.78)	1 (1.43)	10 (14.29)	57 (81.43)

S. 1 y 2: Scores of “Totally disagree/Disagree”; S. 3: Score of “Indifferent”; S. 4 y 5: Scores of “Agree/Totally agree”. Source: The Authors.

**Table 4:** Mean, standard deviation, medians and interquartile range of the Scale with High-Fidelity Clinical Simulation factors in each of the centres.

Factors	Arithmetic Mean ± SD [Median/Interquartile Range] of the Items		
	Centre A	Centre B	Centre C
Factor 1. Simulation utility	3.57 ± 0.71 [4.0/1.0]	3.62 ± 0.59 [4.0/1.0]	3.9 ± 0.55 [4.0/1.0]
Factor 2. Characteristics of cases and applications	3.88 ± 0.61 [4.0/1.0]	3.9 ± 0.47 [4.0/0.0]	4.06 ± 0.55 [4.0/1.0]
Factor 3. Communication	3.79 ± 0.57 [4.0/1.0]	3.39 ± 1.05 [4.0/1.0]	3.86 ± 0.91 [4.0/1.0]
Factor 4. Self-reflection on performance	3.97 ± 0.75 [4.0/1.0]	3.93 ± 0.70 [4.0/1.0]	4.2 ± 0.65 [4.0/1.0]
Factor 5. Increased self-confidence	3.76 ± 0.85 [4.0/2.0]	3.41 ± 0.84 [4.0/1.0]	4 ± 0.85 [4.0/1.0]
Factor 6. Relation between theory and practise	3.6 ± 0.69 [4.0/2.0]	3.53 ± 0.68 [4.0/1.0]	3.7 ± 0.60 [4.0/1.0]
Factor 7. Facilities and equipment	3.79 ± 0.66 [4.0/1.0]	3.35 ± 0.75 [4.0/2.0]	3.75 ± 0.72 [4.0/1.0]
Factor 8. Negative aspects of simulation	3.65 ± 1.06 [4.0/1.0]	3.87 ± 0.86 [4.0/0.0]	4.34 ± 0.83 [4.5/1.0]

Source: the authors.

**Table 5:** Kruskal–Wallis test of the factors according to centre.

Factors	Centre	Average Range	Statistical	*p*	Contrast	Sig.	Difference
1. Simulation utility	A	80.446	9.0582	0.0107	A–B	-	−1.842
	B	82.288			A–C	*	−24.668
	C	105.114			B–C	-	−22.825
2. Characteristics of cases and applications	A	85.2	3.5348	0.1707	A–B	-	1.111
	B	84.088			A–C	-	−14.342
	C	99.542			B–C	-	−15.454
3. Communication	A	91.130	8.4702	0.0144	A–B	-	18.319
	B	72.811			A–C	-	−10.154
	C	101.286			B–C	*	−28.474
4. Self-reflection on performance	A	84.092	7.0577	0.0293	A–B	-	3.847
	B	80.244			A–C	-	−18.950
	C	103.043			B–C	-	−22.798
5. Self-confidence	A	89.938	13.7326	0.0010	A–B	-	21.371
	B	68.566			A–C	-	−15.183
	C	105.121			B–C	*	−36.554
6. Relation between theory and practise	A	88.315	1.2691	0.5301	A–B	-	2.815
	B	85.5			A–C	-	−7.427
	C	95.742			B–C	-	−10.242
7. Facilities and equipment	A	97.853	12.1461	0.0023	A–B	*	30.476
	B	67.377			A–C	-	−0.681
	C	98.535			B–C	*	−31.157
8. Negative aspects of simulation	A	75.593	17.8198	0.0001	A–B	-	−5.339
	B	80.933			A–C	*	−33.406
	C	109.0			B–C	*	−28.066

-: No statistical significance; *: statistical significance; Source: the authors.

## Data Availability

The original contributions presented in the study are included in the article; further inquiries can be directed to the corresponding author.

## Public Involvement Statement

No public involvement was utilized in any aspect of this research.

## Guidelines and Standards Statement

This manuscript was drafted against the Checklist for reporting results using observational descriptive studies as research designs [60].
